# Spatio-Temporal Trends and Risk Factors for *Shigella* from 2001 to 2011 in Jiangsu Province, People's Republic of China

**DOI:** 10.1371/journal.pone.0083487

**Published:** 2014-01-08

**Authors:** Fenyang Tang, Yuejia Cheng, Changjun Bao, Jianli Hu, Wendong Liu, Qi Liang, Ying Wu, Jessie Norris, Zhihang Peng, Rongbin Yu, Hongbing Shen, Feng Chen

**Affiliations:** 1 Department of Epidemiology & Biostatistics, School of Public Health, Nanjing Medical University, Nanjing, Jiangsu, China; 2 Jiangsu Province Center for Disease Control and Prevention, Nanjing, Jiangsu, China; 3 National Center for AIDS/STD Control and Prevention, Chinese Center for Disease Control and Prevention, Beijing, China; Global Disease Detection-Kenya, United States of America

## Abstract

**Objective:**

This study aimed to describe the spatial and temporal trends of *Shigella* incidence rates in Jiangsu Province, People's Republic of China. It also intended to explore complex risk modes facilitating *Shigella* transmission.

**Methods:**

County-level incidence rates were obtained for analysis using geographic information system (GIS) tools. Trend surface and incidence maps were established to describe geographic distributions. Spatio-temporal cluster analysis and autocorrelation analysis were used for detecting clusters. Based on the number of monthly *Shigella* cases, an autoregressive integrated moving average (ARIMA) model successfully established a time series model. A spatial correlation analysis and a case-control study were conducted to identify risk factors contributing to *Shigella* transmissions.

**Results:**

The far southwestern and northwestern areas of Jiangsu were the most infected. A cluster was detected in southwestern Jiangsu (*LLR* = 11674.74, *P*<0.001). The time series model was established as ARIMA (1, 12, 0), which predicted well for cases from August to December, 2011. Highways and water sources potentially caused spatial variation in *Shigella* development in Jiangsu. The case-control study confirmed not washing hands before dinner (*OR* = 3.64) and not having access to a safe water source (*OR* = 2.04) as the main causes of *Shigella* in Jiangsu Province.

**Conclusion:**

Improvement of sanitation and hygiene should be strengthened in economically developed counties, while access to a safe water supply in impoverished areas should be increased at the same time.

## Introduction

*Shigella* is a significant global public health concern, especially in developing countries like the People's Republic of China. *Shigella* deaths have been steadily decreasing over time, but this has come about through markedly reduced fatality rates rather than lower incidence [1]. Currently, a considerable burden of *Shigella* still exists among the younger generations in China [2]. A recent study showed that in Jiangsu Province, China, children under five years old and adults over 60 years old accounted for the greatest proportion of *Shigella* infections [3]. Furthermore, caring for sick children and the elderly can result in the loss of work-days among family members, potentially leading to socioeconomic problems. Due to vaccine innovations that have not yet proven effective, a successful prophylaxis against *Shigella* is currently unavailable [4]. The alarming speed of antibiotic resistance is also complicating treatment with traditional and inexpensive medications [5]. In considering the health consequences of the prevalence of *Shigella*, the inefficacious vaccine and the inadequate therapy options, public awareness of *Shigella* (based on a convincing estimation of *Shigella* disease burden and risk factors) is crucial in controlling transmission.

Spatial and temporal trends are classic methods of estimating disease burden. Statistical models are irreplaceable in forecasting short-term trends and have previously been performed successfully on *Shigella* cases in China [6]. Compared with traditional models such as linear regression or correlation coefficients, autoregressive integrated moving average (ARIMA) models take changing trends, periodic changes and random disturbances in time series into consideration. Earnest et al found that an ARIMA model was easier to fit in terms of the parameters and ran more quickly in forecasting notifiable infectious diseases through time-series models [7]. ARIMA models can eliminate seasonal patterns and are widely applied in predictions for epidemics like hemorrhagic fever with renal syndrome (HFRS), influenza, malaria, etc [8], [9]. At the same time, disease maps produced by geographic information system (GIS) tools can display patchy maps of epidemics [10]. The development of spatial models, which describe spatial autocorrelation as well as geographical trends of epidemics, has also become increasingly popular [11], [12]. Spatial and temporal models alone, however, are insufficient for exploring transmission modes. Risk factors can also be used to determine how interventional work should be conducted. Enteric diseases such as *Shigella* appear as complex epidemics related to changes in biology, socioeconomic status, culture/behavior and environment over space and time [13]. As a result, *Shigella* transmission modes that combine climatic, socioeconomic and human behavioral factors remain worthy of study. In recent years, the issue of climatic impact on health has drawn increasing public attention; Zhang et. al. recently announced that under the current trends of climate change, Years Lost due to Disabilities (YLDs) caused by bacillary *Shigella* would double by 2020 in some parts of China [14]. The climate both directly and indirectly determines the features that lead to transmission by influencing the speed of pathogen variation, the accumulation of susceptible hosts, and other environmental indices. A GIS application can illustrate climatic impact on epidemics through spatial correlation. In addition, a case-control study can help serve to explain the socioeconomic statuses or behaviors associated with *Shigella* transmission. Identification of the geographical risk factors and case-control studies are hoped to concomitantly provide information on the complex transmission modes of *Shigella*.

This is the first research study targeted at the spatial and temporal characteristics of *Shigella* in Jiangsu Province over the last decade (2001 to 2011). The authors aimed to illustrate the spatial variation and temporal trends of *Shigella* through use of GIS and the ARIMA model. Furthermore, the use of GIS coupled with a case-control study design facilitated risk factors analysis. The results and conclusions of this study aim to explore appropriate models for *Shigella* surveillance, identify hotspots and risk factors for infection, and provided evidence-based policy advice for interventions.

## Materials and Methods

### Study Area

Jiangsu Province is located in southeast China between longitudes 116°21′–121°54′E and latitudes 30°46′–35°08′N. Affected by the East Asian monsoon climate, the province has an annual mean temperature between 13.6 and 16.1 degrees Celsius and an annual mean precipitation ranging from 704 to 1250 mm. Jiangsu Province is customarily separated into three geographic regions by the 13 cities of Jiangsu: south (Suzhou, Wuxi, Changzhou and Zhenjiang), middle (Nanjing, Yangzhou, Taizhou and Nantong) and north (Xuzhou, Lianyungang, Suqian, Huai'an and Yancheng). Areas belonging to the same region share similar geographic parameters and economic statuses. These three geographic regions are also collectively composed of 103 counties. In this study, the county is defined as the primary sampling unit.

### Data Resources

In China, a reporting system was established based on the Law on Prevention and Control of Infectious Disease that includes all health-care facilities at village, town, county and city levels [15]. In this study, county-level incidence rates and monthly cases of *Shigella* from 2000 to 2011 were obtained from the reporting system by the Jiangsu Province Center for Disease Control and Prevention (CDC). Climatic and environmental data were collected from the Jiangsu Province Meteorological Bureau and consisted of the annual mean temperature, moisture content, distribution of rivers and lakes, distribution of railways and highways, and Normalized Difference Vegetation Index (NDVI) of Jiangsu Province.

### Data Analysis

#### Spatial variation analysis

Spatial analysis was aimed at detecting geographic variation through use of Geographic Information System (GIS, ArcGIS software, version 9.3 ESRI, Redlands, CA, USA) and SaTscan software (version 9.1.1, Boston, MA, USA).

With the help of GIS, a trend surface analysis was applied to detect scattered observations and geographical anomalies [16]. Estimation of incidence rates of unknown nodes was based on the Ordinary Least Square (OLS) method. This ensured minimized squared deviations by all incidence rates (the dependent variable was denoted as Z). Independent variables were longitudes (denoted as X) and latitudes (denoted as Y).

A spatio-temporal cluster analysis (conducted through SaTScan software) and autocorrelation analysis (conducted through GIS) were both applied to detect clusters of *Shigella* within Jiangsu Province. The spatio-temporal statistical method used cylindrical moving windows to scan inside Jiangsu Province in order to detect clustering areas and years. The base diameter and height of the moving window represented the underlying clustering areas and years, respectively. A Log Likelihood Ratio (*LLR*) method inspected the results by comparing real incidence rates with expected ones [17]. The area confirmed as statistically significant in a Monte Carlo test was defined as the most likely cluster.

At the same time, a local autocorrelation analysis was carried out to identify spatial autocorrelations inside the province. Under autocorrelation theory, the closer two locations approach each other, the more likely they are to impact each other's incidence rates [18]. In this study, the Local Moran's Index (*LM_i_*) and the Local Getis-Ord G index (*LG_i_*) were both applied. The Local Moran's Index helped to classify the autocorrelations into positive and negative ones. If incidence rates had similar high values or low values, they were defined as having positive autocorrelation (represented as High-High or Low-Low autocorrelation). If the attributes held opposing high and low values, they were considered to have negative autocorrelation (represent as High-Low or Low-High autocorrelation). The local Getis-Ord G index, moreover, denoted hotspots among positively autocorrelated areas (*P*<0.05). If the G index identified a county as statistically significant, that county might have higher risks of *Shigella* infection than any other county that was positively autocorrelated.

#### Time-series analysis

Autoregressive integrated moving average (ARIMA) models are known to have better veracity and practicability in describing and forecasting epidemic prevalence [19]. Peng et al previously applied an ARIMA model successfully in confirming *Shigella* transmission with seasonal patterns in China [20]. An ARIMA model is defined as ARIMA (*p*, *d*, *q*), which is decided by components *p*, the order of autoregression; *d*, the degree of difference; and *q*, the order of moving average. Because the monthly data on *Shigella* infections showed seasonal fluctuations, a 12-step finite difference method was initially applied to smooth the temporal sequence. These parameters (*p*, *d*, *q*) were decided by an autocorrelation function (ACF) and a partial autocorrelation function (PACF) [21]. The Ljung-Box test was used to verify the goodness-of-fit of the models and compare them with the Akaike information criterion (*AIC*) to determine the final time series model [22], [23]. It measured the ACF of the residuals at the significance level of *P* = 0.05.

#### Geographic factors detection

The goal for this analysis was to describe *Shigella* development under the influence from each risk factor. In this study, data on the annual mean temperature, moisture content, railway or highway distributions, river or lake distributions and NDVI were obtained from the database of the Jiangsu Province Weather Bureau. Each of the risk factors was overlaid with *Shigella* incidence rates on incidence maps through GIS application. ANOVA analysis was conducted to confirm the correlation with each risk factor. The coefficient of determination (*R^2^*) was used to measure the goodness-of-fit by each risk factor. Furthermore, the risk factors were applied using stepwise regression to establish a multivariate regression model on incidence rates. Risk factors were not included unless *P*≤0.05 in the results of the model. After eliminating collinearity from the factors, the best-fitted model was established as the one with the highest *R^2^* of all models.

#### Case-control study

The researchers also conducted a matched case-control study to investigate associated health or lifestyle behaviors among those exposed to the infection. The case-control study was performed in 29 clinics representing every city of Jiangsu. Cases were diagnosed according to the diagnostic criteria under the Law on Prevention and Control of Infectious Disease of China (patients diagnosed with a microscopic stool examination). Controls were healthy or non-enteric infected patients who were closest in age to the cases (±three years) and lived in the same household with them. In cases for which no control was available in the same household, a person in the neighborhood who met the criteria of a control (closest in age and healthy or non-enteric infected) was selected instead. The study ensured that cases had the same geographical distribution as controls. Each case and control pairing was compared for sex and age. In total, 1200 cases and 1270 controls were included, including 1296 males (53.5%) and 1174 females (47.5%). Statistics ensured no significant difference between cases and controls in sex, age or education.

Data were collected through pre-coded questionnaires. Questions addressed the socioeconomic status of the family, household environment and health-related behaviors. Socioeconomic status of the family was determined by “number of family members” and “family income”. Household environment was determined by “household sanitation”, “drinking water hygiene”, “presence of a separate kitchen or toilet from the rest of the house”, and “frequency of garbage collection”. Health-related behaviors were composed of “frequency of dining out” and “habit of washing hands before dinner or after defecation”.

The case-control study was approved by the ethical committee and IRB (Institutional Review Board) of Nanjing Medical University. *χ^2^* analysis was carried out by SAS 9.0 software on the case-control study. The Odds Ratio (*OR*) of each risk factor estimated the power of association between the risk factor and *Shigella* infection. Point estimation provided a 95% confidence interval (*CI*), which was considered statistically significant at *P* = 0.05 if 1 was not included.

## Results

### Spatial variation analysis

**Figure 1:**
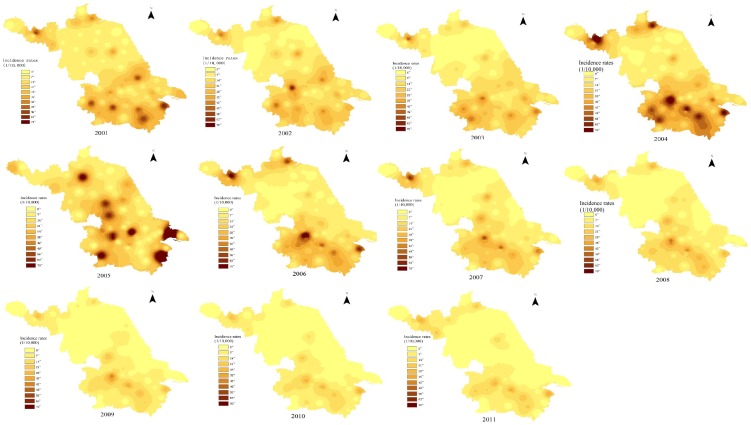
Incidence maps of Shigella in Jiangsu Province from 2001 to 2011.

Incidence maps shown in Figure 1 were made through a GIS application of the Inverse Distance Weighted (IDW) method for interpolation (Figure 1). A deeper color in the maps represented higher incidence rates. Compared with the northern part of Jiangsu, the incidence rates of the southern region have remained at a relatively high level since 2001. The southern region's incidence rate continually increased after that point. Areas with high levels of incidence rates increased (the dark areas expanded) in the southern region. In 2004 and 2005, *Shigella* infection in the south became the most serious. After 2007, the counties with high incidence rates in the southern part gradually shrank to comprise only the southwestern region. According to these maps, the western counties were the most infected areas over the most years within the southern region. Despite this, the incidence rates of the far southeastern region were even higher in 2005. The middle and northern areas of Jiangsu each had relatively low incidence rate levels each year. However, the far northeastern and northwestern counties shared similar incidence rate levels with the most infected southwestern counties in every year except 2005. Comparing the accumulated incidence rates over the 11 years of the study (2001 to 2011) in each county, Xuzhou (in the northwest), Nanjing (in the southwest), Wuxi and Suzhou (in the southeast) had the greatest accumulated incidence rates among all counties, with Nanjing having the highest of the four (346.04 infections per 100,000 residents in 11 years).

#### Trend surface analysis

**Figure 2:**
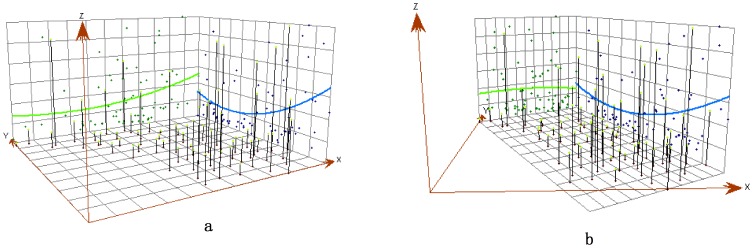
Trend surface graphs of Shigella incidence rates in 2001.

The geographic incidence trends of *Shigella* around Jiangsu Province fluctuated from 2004 to 2006, consistent with the incidence maps (Table 1). The authors chose two locations expressing trends in four directions (Figure 2): the west to east direction (x axis), the south to north direction (y axis) (Figure 2a), the southwest to northeast direction, and the southeast to northwest direction (Figure 2b).

**Table 1:** Trends for Shigella incidence rates.

Year	West-East	South-North	Southwest-Northeast	Southeast-Northwest
**2001**	Increase	U shape	Decrease	U shape
**2002**	Increase	Decrease	Decrease	Decrease
**2003**	Increase	Decrease	Decrease	Decrease
**2004**	No trend	U shape	Decrease	U shape
**2005**	Inverted U shape	Inverted U shape	Inverted U shape	Inverted U shape
**2006**	No trend	U shape	Decrease	U shape
**2007**	No trend	Decrease	Inverted U shape	U shape
**2008**	No trend	Decrease	Inverted U shape	Decrease
**2009**	No trend	No trend	Inverted U shape	Decrease
**2010**	No trend	Decrease	Inverted U shape	Decrease
**2011**	No trend	U shape	Inverted U shape	U shape

Incidence rates increased from west to east in years before 2004, but this trend was less visible after 2004. In 2002, 2003, 2007, 2008 and 2010, *Shigella* incidence rates decreased from the south to the north, but formed a U-shaped trend in another four years (2001, 2004, 2006 and 2011); that is, they decreased from the south to the middle and increased from the middle to the north. Incidence rates decreased when the direction moved from the southwest to the northeast before 2007. For trends in the southeast-northwest direction, in five years (2002, 2003, 2008, 2009 and 2010) the incidence rates rose from northwest to southeast, but in another five (2001, 2004, 2006, 2007 and 2011) they manifested a U-shaped trend. In 2005, trends of all directions were more distinctive than in any other year: they formed an inverted U shape, indicating that southern and northern incidence rates were even lower than those in the middle region.

#### Spatio-temporal cluster analysis and local autocorrelation analysis

Spatio-temporal cluster analysis was applied to observe the incidence rates of *Shigella* from 2001 to 2011 in Jiangsu Province. Incidence rates were aggregated through space and time. A most likely cluster was observed in the southwestern region of the province from 2001 to 2004. This cluster was made up of 30 counties in the southwestern region, including Liyang county, Lishui County, Gaochun County, Jintan County, Yixing County, Jurong County, Danyang County, Changzhou County, Zhenjiang County, Yangzhong County, Wuxi County, Nanjing County, Jiangyin County, Suzhou County, Jingjiang County, Taixing County, Yizheng County, Yanzhou County, Taizhou County, Wujiang County, Jiangdu County, Zhangjiagang County, Changshu County, Jiangyan County, Rugao County, Nantong County, Kunshan County, Gaoyou County, Hai'an County and Taicang County. The cluster window was centered at 31.4205 N, 119.3564 E, in a county named Liyang. The base diameter of the window was 169.96 km, with a Relative Risk (*RR*) of 2.61. The Log Likelihood Ratio for the analysis was 1674.74, *P*<0.001.

Results of the local autocorrelation analysis are shown in Table 2 (Table 2). In 2001, the only detected hotspot was in Suzhou County, but it disappeared in 2002. In 2003, two negatively autocorrelated counties were detected, between which Xuzhou had the higher incidence rate and was surrounded by low incident counties. In 2005, hotspots lying at the southeast region were replaced by hotspots in the southwestern region. In this year, most of the total hotspots were located in the southwestern counties. In 2007, the southeastern hotspots Wuxi and Suzhou were again detected as hotspots, along with other southwestern hotspots. Among all 15 hotspots during the 11 years, Wuxi county and Suzhou County remained hotspots the greatest number of times (six times each). Danyang County, Zhenjiang County and Jurong County were detected as hotspots five, four and two times, respectively. Each of the other 10 counties appeared only once.

**Table 2:** Local autocorrelation analysis for Shigella in Jiangsu Province.

Year	County	*LG_i_ Z* Score	*LGi P* Value	*LM_i_* Index	*LM_i_ Z* Score	*LM_i_ P* Value	Correlation Type
**2001**	Suzhou County	2.80	0.01	<0.01	3.91	<0.01	HIGH-HIGH
**2003**	Tongshan County	1.39	0.16	<0.01	−2.62	0.01	LOW-HIGH
**2003**	Xuzhou County	0.74	0.46	<0.01	−3.12	<0.01	HIGH-LOW
**2004**	Suzhou County	2.45	0.01	<0.01	2.67	0.01	HIGH-HIGH
**2004**	Wuxi County	2.21	0.03	<0.01	2.76	0.01	HIGH-HIGH
**2005**	Siyang County	2.21	0.03	<0.01	2.19	0.03	HIGH-HIGH
**2005**	Danyang County	3.24	<0.01	<0.01	3.49	<0.01	HIGH-HIGH
**2005**	Zhenjiang County	4.86	<0.01	<0.01	4.25	<0.01	HIGH-HIGH
**2005**	Yangzhou County	4.50	<0.01	<0.01	2.66	0.01	HIGH-HIGH
**2005**	Taizhou County	4.20	<0.01	<0.01	6.33	<0.01	HIGH-HIGH
**2005**	Jiangyan County	3.71	<0.01	<0.01	4.68	<0.01	HIGH-HIGH
**2005**	Taixing County	3.63	<0.01	<0.01	3.44	<0.01	HIGH-HIGH
**2005**	Yizheng County	2.11	0.04	<0.01	2.18	0.03	HIGH-HIGH
**2005**	Jiangdu County	4.47	<0.01	<0.01	3.78	<0.01	HIGH-HIGH
**2005**	Jingjiang County	2.39	0.02	<0.01	2.30	0.02	HIGH-HIGH
**2005**	Yangzhong County	4.93	<0.01	<0.01	16.03	<0.01	HIGH-HIGH
**2006**	Danyang County	2.83	<0.01	<0.01	2.73	0.01	HIGH-HIGH
**2006**	Zhenjiang County	2.64	0.01	<0.01	3.98	<0.01	HIGH-HIGH
**2006**	Jurong County	3.25	<0.01	<0.01	4.38	<0.01	HIGH-HIGH
**2007**	Wuxi County	1.98	0.05	<0.01	2.18	0.03	HIGH-HIGH
**2007**	Danyang County	2.50	0.01	<0.01	3.24	<0.01	HIGH-HIGH
**2007**	Zhenjiang County	1.83	0.07	<0.01	2.34	0.02	HIGH-HIGH
**2008**	Suzhou County	2.54	0.01	<0.01	3.17	<0.01	HIGH-HIGH
**2008**	Wuxi County	2.84	<0.01	<0.01	4.32	<0.01	HIGH-HIGH
**2008**	Danyang County	2.35	0.02	<0.01	2.59	0.01	HIGH-HIGH
**2008**	Jurong County	3.05	<0.01	<0.01	2.77	0.01	HIGH-HIGH
**2009**	Suzhou County	2.29	0.02	<0.01	2.50	0.01	HIGH-HIGH
**2009**	Wuxi County	2.97	<0.01	<0.01	4.93	<0.01	HIGH-HIGH
**2009**	Changzhou County	2.38	0.02	<0.01	2.18	0.03	HIGH-HIGH
**2009**	Danyang County	2.74	0.01	<0.01	3.22	<0.01	HIGH-HIGH
**2009**	Zhenjiang County	2.28	0.02	<0.01	2.64	0.01	HIGH-HIGH
**2010**	Suzhou County	2.16	0.03	<0.01	2.27	0.02	HIGH-HIGH
**2010**	Wuxi County	2.63	0.01	<0.01	3.71	<0.01	HIGH-HIGH
**2011**	Suzhou County	2.06	0.04	<0.01	1.98	0.05	HIGH-HIGH
**2011**	Wuxi County	2.81	<0.01	<0.01	4.26	<0.01	HIGH-HIGH
**2011**	Nanjing County	2.14	0.03	<0.01	2.29	0.02	HIGH-HIGH

### Time series analysis

Generally speaking, the number of *Shigella* cases decreased over the years studied. An obvious periodicity by season was observed. The epidemic incidence rates peaked every summer and autumn of the years studied (July to August). Incidence rates decreased from 2001 (25.80 cases per 100,000 residents) to 2003 (19.07 cases per 100,000 residents) and peaked in 2004 (30.60 cases per 100,000 residents). A notable decrease occurred in 2005. In 2006, however, incidence rates rose to a level equal to those in 2001, then kept decreasing after 2006. In 2010, the incidence rate reached its lowest point: 11.46 cases per 100,000 residents. In 2011, the incidence rate was 12.23 cases per 100,000 residents, less than half of what it was in 2001.

**Figure 3:**
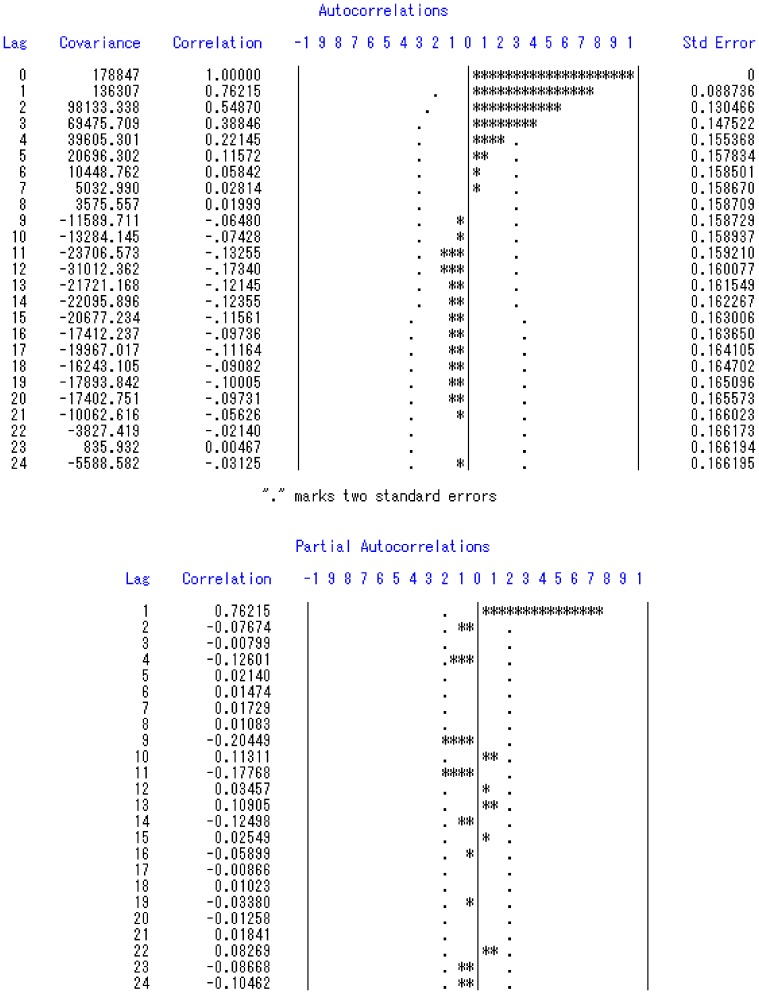
Results of ACF and PACF for time series analysis.

Analysis was performed by SAS with significance level *P* = 0.05, based on the monthly data of Jiangsu Province from January 2001 to July 2011. After the 12-step difference operation, the trend sequence was flat. Results of the autocorrelation function (ACF) and partial autocorrelation function (PACF) suggest that it was proper to establish an AR(1) model with no constant included (Figure 3). The AIC turned out to be 1787.77. The autocorrelation check for the residuals ensured *P*>0.05 for each lag, which indicated the model fitted well for the data (Table 3). The final model was established as ARIMA (1, 12, 0).

**Table 3:** Autocorrelation check of residuals in time series analysis.

To lag	χ^2^	DF	*p*	Autocorrelations					
6	2.65	5	0.75	0.05	−0.02	0.07	−0.08	−0.07	−0.04
12	15.11	11	0.18	−0.03	0.15	−0.14	0.08	−0.04	−0.19
18	18.23	17	0.37	0.09	−0.03	−0.03	0.06	−0.07	0.05
24	21.71	28	0.54	−0.03	−0.08	0.02	0.02	0.12	−0.03

**Figure 4:**
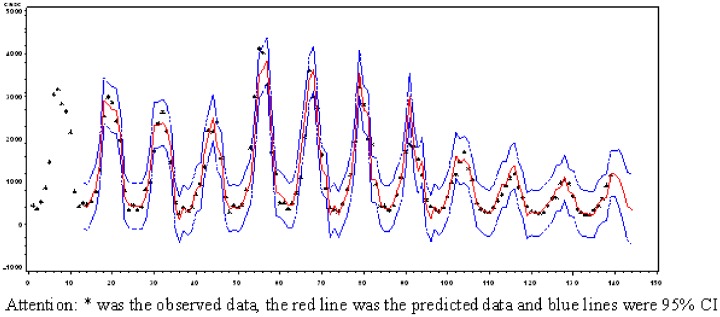
Prediction for Shigella cases from August to December in 2011.

Based on the ARIMA model, the study attempted to predict cases from August to December, 2011 (Table 4). When compared with the observed data, the predicted data agreed very closely with the observed data (Figure 4). Predicted monthly numbers of cases from August to December 2011 were 1172.97, 1039.40, 717.16, 382.24 and 292.06, respectively.

**Table 4:** Forecasts for variable cases of 2011.

Observations	Forecast	Standard Error	95% Confidence Limits	
August	1172.97	275.24	633.51	1712.43
September	1039.40	346.61	360.16	1718.84
October	717.16	382.28	−32.10	1466.41
November	382.24	401.71	−404.99	1169.68
December	292.06	412.66	−516.75	1100.86

### Risk factor detection

#### Geographic factors detection

**Figure 5:**
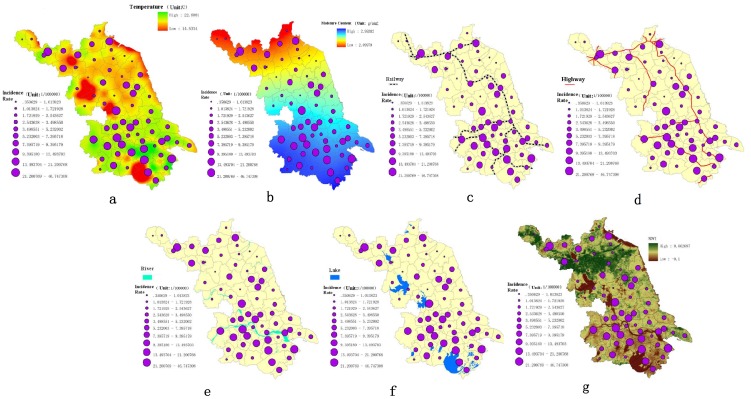
Layout charts concerning risk factors and incidence rates.

Layout charts of the univariate analysis were displayed (Figure 5a–5g). The climatic factors considered were annual mean temperature and moisture content. Distances to railways, highways, lakes, and rivers, as well as NDVI, comprised the environmental factors. A redder color indicates a lower mean temperature (Figure 5a). The moisture content was between 2.10 to 2.99 g/cm^2^ (Figure 5b). Incidence rates tended to rise in exponential trends with both annual mean temperatures and moisture content. However, the trends were not obvious with *R^2^* equal to 0.16, *P* = 0.001 (annual mean temperature) and *R^2^* equal to 0.14, *P* = 0.002 (moisture content), respectively.

Buffer zones were established for each railway, highway, river or lake to estimate distances from them (Figure 5c–5f). Incidence rates increased with decreasing distances to railways (*R^2^* = 0.99, *F* = 576.61, *P*<0.001), rivers (*R^2^* = 0.98, *F* = 143.68, *P*<0.001) and lakes (*R^2^* = 0.99, *F* = 11411.51, *P*<0.001) and they peaked at an average of 3 km from the rivers. However, they also peaked at an average 15 km from the highways (*R^2^* = 0.99, *F* = 2582.18, *P*<0.001). No convincing evidence of an association between the NDVI and the incidence rate was observed, with *R^2^* = 0.08, *P* = 0.128 in spring, *R^2^* = 0.04, *P* = 0.118 in summer, *R^2^* = 0.05, *P* = 0.367 in autumn and *R^2^* = 0.17, *P*<0.001 in winter (Figure 5g).

Based on the above results, risk factors of distances from railways, highways, rivers and lakes were chosen for stepwise regression. However, railway distance was excluded because *P* = 0.26 in the first step of regression. A collinearity diagnosis ensured no collinearity existed. The final model was comprised of all three factors, with the highest coefficient of determination *R^2^* being 0.53, *R* = 0.73. It was established as:
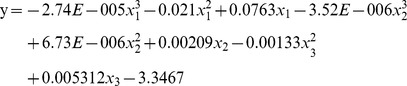
where y represents the *Shigella* incidence rate, and *x_1_*, *x_2_* and *x_3_* stand for distances to the river, the lake and the highway, respectively. ANOVA analysis confirmed *F* = 140.56, *P*<0.001.

#### Case-control study

Results of the univariate analysis in the case-control study are listed (table 5). According to the results, controls tended to have a higher family income (*χ^2^* = 35.71, *P*<<0.011) and fewer family members (*χ^2^* = 33.77, *P*<<0.011) than cases. Also, controls were more likely to have good household hygiene (*χ^2^* = 59.12, *P*<<0.011), use a separate kitchen (*χ^2^* = 25.47, *P*<<0.011) and toilet (*χ^2^* = 65.52, *P*<<0.011) from the rest of the house, and have access to safe drinking water (*χ^2^* = 74.54, *P*<<0.011). Health-related behaviors such as washing hands before dinner (*χ^2^* = 240.62, *P*<<0.011) and after defecation (*χ^2^* = 131.41, *P*<<0.011), and the frequency of treating household garbage (*χ^2^* = 14.44, *P*<<0.011) also differed between cases and controls.

**Table 5:** Univariate case-control analysis between Shigella infection and risk factors.

Variable	Groups	Cases N	Controls N	χ^2^	*P*	*OR*	95%CI	
Family members	More than six	434	327	33.77	<<0.011	1		
	Between four and six	387	441			1.51	1.24	1.84
	Less than four	379	502			1.75	1.44	2.13
Family income per capita	<2000 Yuan/Month	454	371	35.71	<<0.011	1		
	2000∼4000 Yuan/Month	425	421			1.21	1.01	1.47
	>4000Yuan/Month	321	478			1.82	1.49	2.22
Household sanitation	Not good	671	531	59.12	<<0.011	1		
	Good	529	739			1.76	1.51	2.07
Safety of drinking water supply	Not Good	803	632	74.54	<<0.011	1		
	Good	397	638			2.04	1.73	2.40
Separate kitchen	No	669	579	25.47	<<0.011	1		
	Yes	531	691			1.50	1.28	1.76
Separate toilet	No	718	553	65.52	<<0.011	1		
	Yes	482	717			1.93	1.64	2.26
Disposing of household garbage	Daily/every other day	563	693	14.44	<<0.011	1		
	More	637	577			0.73	0.63	0.86
Washing hands after defecation	No	677	991	131.41	<<0.011	1		
	Yes	523	279			0.36	0.30	0.42
Washing hands before dinner	No	799	449	240.62	<<0.011	1		
	Yes	401	821			3.64	3.08	4.30

Univariate analysis illustrated that socioeconomic status (number of family members and family income) was negatively associated with *Shigella* infection. In the study, 379 cases and 502 controls reported having fewer than four members in their households (*OR*, 1.75 [*CI*, 1.44–2.13]). A total of 434 cases and 327 controls reported having more than six family members (*OR*, 1.51 [*CI*, 1.24–1.84]). With regard to family income, 454 cases and 371 controls belonged to the lowest group level of ‘less than 2000 Yuan/Month’ (*OR*, 1.21 [*CI*, 1.01–1.47]). Household environment also turned out to impact *Shigella* infection. This was observed in people not having a separate kitchen or toilet from the rest of the house, with *OR*'s being 1.50 and 1.93, respectively. “Poor household hygiene” or “lack of a safe drinking water supply” were additional risk factors for *Shigella* infection (in Table 5). Washing hands before dinner was strongly associated with *Shigella* infection, with *OR* = 3.64, *CI* [3.08–4.03]. Furthermore, the authors also detected that disposing of household garbage every day or every other day potentially protected against the chance of *Shigella* infection (*OR*, 0.73 [*CI*, 0.63–0.86]).

## Discussion

The primary aim of this research was to illustrate spatial and temporal trends of *Shigella* in Jiangsu Province. Incidence maps indicated the far western areas of the southern and northern regions were most vulnerable to *Shigella* infections. Although infections in the southern region continually accounted for the greatest proportion, the high incident area of the northern region expanded its range in the latter years of the decade (after 2004). The total incidence rate was observed decreasing in Jiangsu Province, which was confirmed by the time-series analysis, but this decrease in total incidence rates may obscure the fact that some parts of the northern region may have had increases in *Shigella* incidence rates. Trend surface analysis confirmed the conclusions of the incidence maps. In contrast with the continuously decreasing trend in the south-to-north direction, the trend curves changed to a U shape in some years (2001, 2004, 2006 and 2011). This indicated that northern incidence rates might have approached parity with those of the southern region.

Suzhou and Wuxi were the two most prominent counties for *Shigella* transmissions in the southeastern region; they were identified as hotspots the greatest number of times among all counties (in 2001, 2007, 2008, 2009, 2010, 2011). However, a cluster was also detected at the southwestern part of Jiangsu from 2001 to 2004. The hotspots detected in the autocorrelation analysis indicated that hotspots were aggregated in the southwestern region after 2005. This indicates that the southwestern region may be becoming a new target for *Shigella*. Notable fluctuations also appeared from the years 2004 to 2006. The ARIMA model confirmed these fluctuations and successfully established a predictive model concerning the time series. Researchers have previously found that the ARIMA model was very effective and reliable in providing decision makers with clear indications of variability among future observations [24]. Establishment of the ARIMA model could help to forecast *Shigella* developments, as well as warn epidemiological monitors of potential abnormalities in the future.

Spatial and temporal trends functioned as a first step to determine pathways for epidemic development and discover potential target areas. Another important aim of this study was to identify potential risk factors. Many researchers have confirmed that geographical factors such as ambient temperatures and relative humidity are driving *Shigella* transmissions [25], [26]. However, these conclusions were mostly based on temporal analyses using monthly data. In this article, geographical risk factors were obtained to explore the geographical reasons causing spatial variability among counties. According to the results, temperature and moisture content were not the primary reasons for spatial variations in incidence rates in Jiangsu Province; the distances to railways, highways, rivers and lakes contributed substantially more. Transportation may have led to more frequent interactions among cities or provinces, which concurrently increased the chances of *Shigella* transmission. Since railways and highways are the two main methods of long distance travel in Jiangsu, this indicates that sanitation policies around railways or highways should be well enforced.

The stepwise regression model helped to correlate results with environmental conditions. Study results confirmed that distances to highways, rivers and lakes facilitated *Shigella* transmission, and also indicated the importance of reducing *Shigella* infections transmitted through water systems. Since water sources were the leading reason for spatial variation, improving the imbalanced quality of water supplies in the northern and southern regions should be the next target for *Shigella* prevention. The case-control study confirmed a safe water supply as an important protective factor against *Shigella* transmission. Inadequate water and sanitation not only increased the chances of diarrheal infection, but were also associated with poorer child health status in the long term [27]. In the less developed northwestern areas of Jiangsu Province, the sanitation and hygiene issues drew the authors' greatest concern. The penetration of access to a safe water supply was much lower here than in the southern and middle regions; in 2009, Zhu et. al. announced that in Xuzhou and Lianyungang (two cities of northern Jiangsu), the penetration levels of access to a safe water supply were only 37.3% and 63.2%, respectively (compared to over 92% in other regions) [28]. Furthermore, the rates of treatment of domestic garbage in the northern region were only about one-sixth those in the southern region. Lack of sanitation protection in the northern counties may have increased the transmission of enteric diseases like *Shigella*. Garbage disposal and hand washing have previously been identified as two protective behavioral factors preventing *Shigella* transmission; Rego et. al. declared that children exposed to garbage in their environment were nearly four times more likely to contract diarrhea [29]. Given the presence of organic waste in family garbage, the surrounding environment is prone to attracting disease vectors. This increases the chance of young children being exposed to pathogens through playing with garbage, which presents a challenge to maintaining good family hygiene.

In the case-control study, a higher family income and fewer family members both indicated better socioeconomic status. This group also had a lower incidence rate. In 2008 Ferrer et al. confirmed economic factors as one of the factors influencing enteric burden [30]. Low family income may have predisposed families to a lower nutritional status and a lower awareness of hygiene, thus leading to a higher probability of disease. Also, a crowded environment, in which household hygiene is always hard to enforce, would facilitate person-to-person transmission. However, when compared with the conclusions from the case-control study, socioeconomic factors worked differently on *Shigella* transmissions when pointing to regional variations. The highly developed southern region of Jiangsu apparently had more infections than the middle and northern regions. The southern region of Jiangsu, including Suzhou and Wuxi, currently attracts large amounts of migrants due to its prosperous economic development. Living in a transitory environment and having a precarious lifestyle, the migrant population might not have prioritized food safety and sanitation issues, which made enteric infection the largest threat to their health status. At the same time, the rapid economic development brought a large expansion in business activity and increased the frequency of dining outside of the home. It also increased the possibility of exposure to *Shigella* dysenteriae from polluted food and drinking water. These phenomena brought on by economic development may have led to the high incidence level of the southern areas.

As is well-known, macroscopic descriptions or analyses can easily lead to ecological fallacy problems. Discussions about geographic risk factors in this study were carried out province-wide and conclusions were made based on county-level groups of infections. However, individuals might behave differently than the groups did. As mentioned above, economic problems affect individuals and groups in different ways. In order to avoid a possible ecological fallacy, the case-control study, with its more microscopic focus on individuals, provided supplementary explanations for geographical risk factors, especially regarding access to a safe water supply and other socioeconomic factors. Both the macroscopic and microscopic discussions were combined in this research to analyze geographical risk factors and behavior-related risk factors. In this way, the authors hope that conclusions based on the results might be more scientifically robust than a simple one-way analysis.

Based on these results, future interventions should focus on different points addressing the transmission patterns and underlying risk factors of different regions. Improvements in sanitation and hygiene should be strengthened at the county level, while access to safe water supplies in impoverished areas should be increased at the same time. Furthermore, strengthening public education towards prevention of *Shigella* should be incorporated with interventional policies to eliminate or reduce *Shigella* infection, such as advocating washing hands correctly and regularly, or using hygiene facilities in the household. Srtina et al. concluded that hygienic behaviors were associated with a family's individual predisposition rather than the neighborhood's [31]. In this way, emphasizing efforts at the domestic level to the application of hygiene facilities is of great importance.

Although this study was aimed at detecting spatial variations and causal risk factors, as well as establishing an ARIMA model for surveillance, the limitations of this research must also be acknowledged. Among all 11 years, the incidence rate was found to increase from 2001 to 2004 but hotspots continued to exist even after 2005, and some of them appeared only once. There is a possibility that some hotspots might have been detected by chance, in which case more surveillance work should be carried out around the province to monitor these hotspots or potential risk clusters. GIS is powerful in predicting and visualizing models combining risk factors, and is gradually being applied more and more to explore epidemic risk factors in recent years [32], [33]. However, in spite of the statistic estimations, visualized incidence maps may easily lead to specious conclusions. In addition, limited data resources make it hard to widely explore environmental risk factors. Therefore the results in this study should be supported by continuous health surveillance programs to see how they function under real-world conditions.
